# Mesenchymal Stromal Cells Derived Extracellular Vesicles Ameliorate Acute Renal Ischemia Reperfusion Injury by Inhibition of Mitochondrial Fission through miR-30

**DOI:** 10.1155/2016/2093940

**Published:** 2016-10-05

**Authors:** Di Gu, Xiangyu Zou, Guanqun Ju, Guangyuan Zhang, Erdun Bao, Yingjian Zhu

**Affiliations:** ^1^Department of Urology, Shanghai General Hospital, School of Medicine, Shanghai Jiao Tong University, Shanghai 200080, China; ^2^Department of Urology, Shanghai Children's Medical Center Affiliated to Shanghai Jiao Tong University School of Medicine, Shanghai 200127, China; ^3^Department of Urology, Zhongda Hospital, School of Medicine, Southeast University, Nanjing 210009, China; ^4^Department of Urology, Shanghai Xinhua Hospital Affiliated to Shanghai Jiao Tong University School of Medicine, Shanghai 200092, China

## Abstract

*Background*. The immoderation of mitochondrial fission is one of the main contributors in ischemia reperfusion injury (IRI) and mesenchymal stromal cells (MSCs) derived extracellular vesicles have been regarded as a potential therapy method. Here, we hypothesized that extracellular vesicles (EVs) derived from human Wharton Jelly mesenchymal stromal cells (hWJMSCs) ameliorate acute renal IRI by inhibiting mitochondrial fission through miR-30b/c/d.* Methods*. EVs isolated from the condition medium of MCS were injected intravenously in rats immediately after monolateral nephrectomy and renal pedicle occlusion for 45 minutes. Animals were sacrificed at 24 h after reperfusion and samples were collected. MitoTracker Red staining was used to see the morphology of the mitochondria. The expression of DRP1 was measured by western blot. miR-30 in EVs and rat tubular epithelial cells was assessed by qRT-PCR. Apoptosis pathway was identified by immunostaining.* Results*. We found that the expression of miR-30 in injured kidney tissues was declined and mitochondrial dynamics turned to fission. But they were both restored in EVs group in parallel with reduced cell apoptosis. What is more, when the miR-30 antagomirs were used to reduce the miRNA levels, all the related effects of EVs reduced remarkably.* Conclusion*. A single administration of hWJMSC-EVs could protect the kidney from IRI by inhibition of mitochondrial fission via miR-30.

## 1. Introduction

Renal ischemia reperfusion injury (IRI) is a primary cause of acute kidney injury (AKI) and often occurs in the context of renal transplantation, sepsis, and shock [1, 2]. When the injury is severe, incomplete renal recovery could lead to proliferation of fibroblasts and excessive deposition of extracellular matrix [3]. Therefore, the patients who have AKI are at high risk for the development of end-stage renal disease [4, 5].

Among multiple pathogenetic mechanisms, such as inflammatory response, cell apoptosis, and oxidant injury [1], emerging evidence has suggested a role of mitochondrial dynamics in IRI [6]. Mitochondria are a class of dynamic organelles that constantly undergo fission and fusion [7]. Under intra- or extracellular stresses, mitochondrial dynamics is shifted to fission state, leading to mitochondrial fragmentation and cell apoptosis [7, 8]. Dynamin-related protein 1 (DRP1), a key regulator of mitochondrial fission [7, 8], has been reported to be activated rapidly following AKI [6, 8, 9]. Notably, the inhibition of DRP1 via a dominant-negative mutant or a pharmacologic inhibitor midivi-1 could block mitochondrial fragmentation and protect kidneys against ischemia or cisplatin nephrotoxic AKI [6, 10]. These studies indicate that DRP1 may be an important therapeutic target for ischemia AKI.

MicroRNAs (miRNAs) are small conserved RNA molecules with potential roles in regulation of gene expression at posttranscriptional levels [11]. After binding to target mRNAs, miRNAs form a complex with them and reduce their protein levels by degrading the mRNA or suppressing the translation of the target gene [11, 12]. miRNAs control a wide array of biological processes including cell proliferation, apoptosis, and stress response [13]. miRNAs are involved in the pathogenesis of kidney diseases and it might be possible to manipulate miRNA to achieve therapeutic effects [14, 15].

Mesenchymal stromal cells (MSCs) were extensively investigated for their renal reparative property via paracrine/endocrine mechanisms [16, 17]. Extracellular vesicles (EVs) derived from MSCs were described as a novel pathway of cell-to-cell interaction and a crucial point of endocrine [18]. These membranous structures deliver exogenous functional mRNAs and micro-RNAs sequences to target cells and protect kidney from IRI [15, 19]. However, fewer researches of EVs focus on their miRNA regulation function. Given that miR-30 has been reported to regulate mitochondrial fission through the DRP1 pathway on cardiomyocytes [20], we hypothesized that human Wharton Jelly MSCs (hWJMSCs) derived EVs may be involved in the modulation of mitochondrial fission via miR-30, thereby protecting kidney from IRI.

The aim of the present study was to investigate possible therapeutic mechanism of hWJMSC-EVs. We demonstrated that hWJMSC-EVs may ameliorate acute renal IRI by inhibition of mitochondrial fission via miR-30.

## 2. Experimental Procedures

### 2.1. Cell Culture

Fresh human umbilical cords that are usually discarded after delivery were obtained with the written consent of the parents. This experiment was approved by the Research Ethics Committee at Shanghai General Hospital affiliated to Shanghai Jiao Tong University (Permit number: 2013KY018). Human umbilical cords were delivered and stored in cold Hank's balanced salt solution (Sigma-Aldrich, St. Louis, MO, USA) and then cellular isolation started within 4 h. The hWJMSCs were isolated and identified as described previously [21]. Briefly, with the elimination of umbilical cord vessels, mesenchymal tissues were cut into 1 mm^3^ pieces and then stuck to the substrate of culture plates individually, followed by the addition of low-glucose Dulbecco Modified Eagle's Medium (DMEM, Gibco BRL Co., USA) containing 10% fetal bovine serum (FBS, Gibco BRL Co., USA) at 37°C in a humidified atmosphere with 5% CO_2_. The medium was changed every two days. After about two weeks' culture, the adherent cells were harvested with 0.25% trypsin (Gibco BRL Co., USA) treatment and subcultured. Only cells from three to six passages were used for further experiments.

Renal tubular epithelial cells were isolated from SD rat (80 to 100 g) kidney. Cortical tissues were minced into 1 mm^3^ and digested with 0.1 collagenase I (Gibco BRL Co., USA) and centrifuged in 32% Percoll medium to purify proximal tubular cells. Cells were then plated in collagen-coated dishes and maintained in DMEM/F-12 medium supplemented with 10% FBS.

### 2.2. Transfection of miRNA Antagomir

The antagomirs designed to inhibit the expression of endogenous miR-30 members and Antagomir Negative Control were obtained from Ribobio. MSCs were cocultured with the antagomir at a concentration of 50 nM for 24 h. After that, we changed the culture solution for another 24 h before use.

The sequence of miR-30b antagomir is 5′-AGCUGAGUGUAGGAUGUUUACA-3′; miR-30c antagomir is 5′-GCUGAGAGUGUAGGAUGUUUACA-3′; miR-30d antagomir is 5′-CUUCCAGUCGGGGAUGUUUAGA-3′.

### 2.3. Isolation of EVs

EVs were obtained from supernatants of hWJMSCs as was previously described [21, 22]. Briefly, hWJMSCs were cultured in DMEM without FBS and with added 0.5% bovine serum albumin (BSA) (Sigma-Aldrich) overnight. The viability of the cell cultured overnight was >99% as detected by trypan blue exclusion and no apoptotic cells were detected by terminal transferase-mediated dUTP nick-end labeling (TUNEL) assay. The conditioned medium was collected and stored at −80°C. The medium was centrifuged at 2,000 ×g for 20 minutes to remove debris and then ultracentrifuged at 100,000 ×g in a SW41 swing rotor (Beckman Coulter, Fullerton, CA, USA) for one hour at 4°C. EVs were washed once with serum-free M199 (Sigma-Aldrich) containing 25 mM HEPES (pH = 7.4) and submitted to a second ultracentrifugation in the same conditions. EVs were stored at −80°C for the experiments. To quantify the protein content, the Bradford protein assay kit (P0006, Beyotime Institute of Biotechnology, China) was used in the Bradford assay. We quantified it indirectly according to 5 × 10^5^ MSCs releasing approximately 100 *μ*g EVs overnight. RNA extracted from EVs by use of TRIZOL reagent was analyzed by spectrophotometer.

### 2.4. ATP Depletion Treatment

Renal tubular epithelial cells were treated with 10 mM azide in glucose-free and glutamine-free DMEM (Gibco, USA) for 2 hours. Then, the medium was changed to normal DMEM/F12 for 2-hour reperfusion. In EVs treatment group, 20 *μ*g EVs were added to the medium for reperfusion. After the incubation, cells were fractionated or fixed for the next analysis.

### 2.5. Animal Models of Unilateral Renal IRI

All work involving animals was done in accordance with the animal use protocol enacted by the Institutional Animal Care and Use Committees of School of Medicine, Shanghai Jiao Tong University. Animal models were performed in male SD rats (180 to 200 g) by right kidney resection and left ischemia for 45 minutes. Sham-treated animals were treated in a similar manner, except that the renal vessels were not clamped. A total of 100 *μ*g EVs in 1 mL of vehicle (M199, Gibco BRL Co., USA) or a total of 1 mL of vehicle only was administered via caudal vein immediately after reperfusion. The animals were separated into different groups according to different therapeutic procedures. We used at least six rats for each group: (1) sham (*n* = 6); (2) vehicle (*n* = 6); (3) EVs (*n* = 6); (4) EVs + antagomir control (*n* = 6); (5) EVs + antagomir miR-30b (*n* = 6); (6) EVs + antagomir miR-30c (*n* = 6); (7) EVs + antagomir miR-30d (*n* = 6); (8) EVs + antagomir miR-30b/c/d (*n* = 6). The rats were sacrificed at 24 h. Blood and kidney samples were collected and submitted for corresponding examination.

### 2.6. Mitochondrial Staining and Imaging

Renal tubular epithelial cells were plated onto the coverslips coated with 0.01% poly-L-lysine. After treatment, they were stained for 20 min with 0.02 mM MitoTracker Red CMXRos (Molecular Probes). Then, they were fixed with paraformaldehyde for another 20 min and stained with DAPI. Mitochondria were imaged using a laser scanning confocal microscope (LEICA SP8). For each sample, several random fields of cells (≥100 cells per field) were evaluated for mitochondrial morphology.

### 2.7. Renal Function

Blood samples were obtained for measurement of plasma BUN and creatinine. Serum creatinine and BUN were measured by a Beckman Analyzer II (Beckman Instruments, Inc.).

### 2.8. Immunohistochemistry and TUNEL Assay

One portion of the renal tissue was fixed in 4% paraformaldehyde and embedded in paraffin. 4 um thick sections were labeled with mouse antibody to rat caspase 9 (dilution 1 : 250; Roche) followed by horseradish peroxide- (HRP-) conjugated secondary antibody using diaminobenzidine (DAB) reagents as substrates and then counterstained with hematoxylin. Under 400x magnification, scoring for caspase 9-positive cells was carried out by counting the average number of positive tubular cells in 30 random fields from randomly chosen kidney sections for each animal (*n* = 6 rats, each group).

TUNEL assay was performed to evaluate apoptosis in renal tissues using the In Situ Cell Death Detection Kit from Roche Applied Science. Renal tissues were fixed with 4% paraformaldehyde and paraffin embedded. Tissue sections of 4 *μ*m were exposed to a TUNEL reaction mixture containing terminal deoxynucleotidyl transferase and nucleotides, including tetramethylrhodamine-labeled (TMR-labeled) dUTP. The slides were examined by fluorescence microscopy. Kidney sections were screened for tubular cells of positive nuclei under 400x magnification. The apoptotic score was achieved by counting the average number of positive nuclei in 30 pictures from randomly chosen kidney sections for each animal (*n* = 6 rats, each group).

### 2.9. Mitochondria Isolation

Mitochondria were isolated using a mitochondria isolation kit from Thermo Fisher (USA) according to the manufacturer's instructions. Briefly, kidney homogenate was added to Mitochondria Isolation Reagent. Through several times of differential centrifugation, we collected the mitochondria for next analysis. Cytoplasmic protein was isolated using the RIPA Lysis and Extraction Buffer from Thermo Fisher (USA). Protein was denatured and analyzed by western blotting.

### 2.10. Western Blot

Protein concentration was measured with BCA Protein Assay; 30 *μ*g of total protein was electrophoresed on a 5% to 15% SDS-PAGE gel and then transferred onto nitrocellulose membranes (Millipore, USA). Membranes were blocked in 5% nonfat milk in TBS containing 0.1% Tween 20 for 1 h at room temperature, and then each membrane was incubated with a rabbit antibody to rat DRP1 (dilution 1 : 1,000; CST) or a rabbit antibody to voltage-dependent anion channel (VDAC) (dilution 1 : 1,000; CST) overnight at 4°C. VDAC antibody served as a loading control. After being washed in TBST, each membrane was incubated for one to two hours with a secondary antibody conjugated by peroxidase at 37°C, detected by ECL reagent (Millipore, USA). The density of each band was analyzed by Image-Pro Plus 6.0 software. The densitometry data of DRP1 was normalized to VDAC and relative DRP1 levels were obtained for each group.

### 2.11. Quantitative Real-Time PCR

Total RNA was isolated from hWJMSC-EVs and kidney samples by TRIZOL (Invitrogen, Carlsbad, CA, USA) extraction method. RNA concentration and RNA integrity were determined by DU800 UV/Vis Spectrophotometer (Beckman Coulter, CA, USA). Total RNA was processed to cDNA by reverse transcription using the TRIO Thermocycler (Biometra, Germany). qRT-PCR was performed with a SYBR green-based PCR master mix kit (Takara, Shiga, Japan) on a Mx3000p™ (STRATAGENE, USA). The levels of miR-30 family members analyzed by qRT-PCR were normalized to that of U6. MiR-30 levels refer to the fold change of each experimental group.

The sequences of miR-30b primers were Forward: 5′-TGTAAACATCCTACACTCAG-3′; Reverse: 5′-AACTGGTGTCGTGGAG-3′; RT: 5′-CTCAACTGGTGTCGTGGAGTCGGCAATTCAGTTGAGAGCTGAGT-3′. The sequences of miR-30c primers were Forward: 5′-AAACATCCTACACTCTCAG-3′; Reverse: 5′-TGGTGTCGTGGAGTC-3′; RT: 5′-CTCAACTGAATTGCCGACTCCACGACACCAGTTGAGGCTGAGAG-3′. The sequences of miR-30d primers were Forward: 5′-TAAACATCCCCGACTG-3′; Reverse: 5′-AACTGGTGTCGTGGAG-3′; RT: 5′-CTCAACTGGTGTCGTGGAGTCGGCAATTCAGTTGAGCTTCCAGT-3′.

### 2.12. Statistical Analysis

Data were expressed as the means ± standard deviation (SD) from three independent experiments. Statistical analyses were performed using SPSS v19.0. A value of *P* < 0.05 was considered to be statistically significant.

## 3. Results

### 3.1. hWJMSC-EVs Alleviate Mitochondrial Fragmentation and DRP1 Activation in the Model of IRI

**Figure 1:**
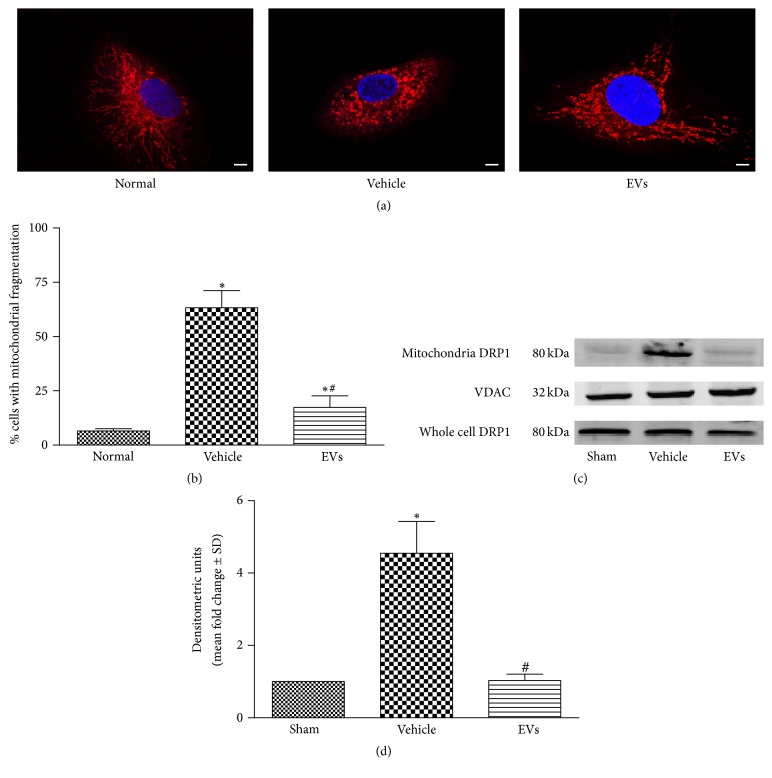
hWJMSC-EVs alleviate mitochondrial fragmentation and DRP1 activation in the model of IRI in vitro and in vivo. (a) Two hours after the treatment with azide, the renal tubular epithelial cells were stained with MitoTracker Red. The morphological alterations of mitochondria in renal tubular epithelial cells were imaged using a laser scanning confocal microscope (the original magnification is 630x). Bar = 5 um. (b) The percentage of cells undergoing mitochondrial fission. (c, d) Mitochondrial DRP1 expression in kidney tissues was measured by western blot analysis at 24 hours after sham, IRI, or EVs treatment. VDAC antibody served as a loading control. Data in (b) and (d) are presented as mean ± SD; *n* = 6; ^*∗*^ *P* < 0.05, versus sham; ^#^ *P* < 0.05, versus vehicle.

We previously demonstrated that hWJMSC-EVs have protective effect on renal IRI [21, 22]. To examine mitochondrial morphological alterations under this condition, MitoTracker Red was used to label mitochondria with red fluorescence after the rat renal tubular epithelial cells were subjected to azide induced 2 h ATP depletion and 2 h reperfusion to model IRI in vitro. 20 *μ*g of EVs was added to the medium for reperfusion in EVs treatment group. Then, the cells were imaged with confocal laser scanning microscope. As shown in Figure 1(a), mitochondria in normal cells were filamentous or of thread-like appearance and were often interlinked to form a network. Upon azide treatment, the mitochondrial network broke down and the mitochondria were fragmented into numerous round fragments of varying size. However, the EVs treatment preserved the mitochondrial network morphology. Quantification of mitochondrial fragmentation is shown in Figure 1(b). EVs treatment mitigates the mitochondrial fragmentation caused by IRI.

As DRP1 is one of the most important determinants in mitochondria morphology, immunoblot analysis of DRP1 in rat kidney in mitochondria fractions and whole cell lysis was performed. We demonstrated that DRP1 activated and translocated to mitochondria 24 hours after IRI and EVs treatment blocked the activation (Figures 1(c) and 1(d)). We observed the lower expression of mitochondrial DRP1 in EVs treatment group. These data indicate that IRI activates DRP1 and triggers the mitochondrial fragmentation. However, both of them are prevented by EVs treatment, which may be one of the mechanisms that provide therapeutic effects for renal IRI.

### 3.2. HWJMSC-EVs Deliver and Restore the Expression of miR-30 in Injured Rat Kidney

**Figure 2:**
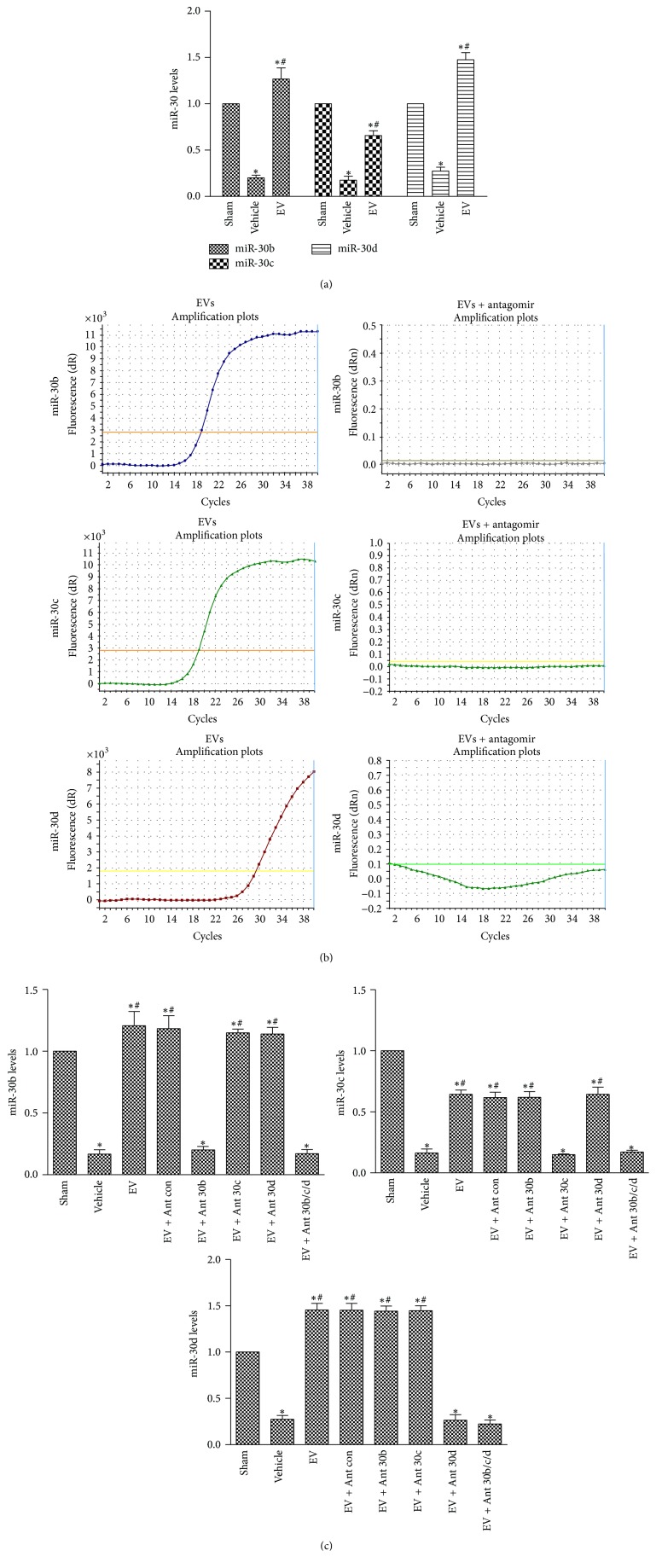
hWJMSC-EVs deliver and restore the expression of miR-30 in injured renal tubular epithelial cells. (a) Representative quantitative reverse transcriptase- (qRT-) PCR analysis for miR-30b/c/d in kidney tissues 24 hours after sham, IRI, or EVs treatment. (b) qRT-PCR analysis of miR-30b/c/d content in EVs derived from MSCs cultured with vehicle alone (wild-type) or subjected to miR-30b/c/d antagomir. (c) miR-30b/c/d levels in kidney in different experimental conditions, including sham, vehicle, EVs, and miR-30b/c/d antagomir-treated EVs group. Data in (a) and (c) are presented as mean ± SD; *n* = 6; ^*∗*^ *P* < 0.05, versus sham; ^#^ *P* < 0.05, versus vehicle.

We next explored the miR-30 expression of rat kidney during IRI in vivo. Quantitative reverse transcriptase polymerase chain reaction (qRT-PCR) showed that IRI caused lower expression of miR-30b, miR-30c, and miR-30d (miR-30a and miR-30e did not exist in rat kidney) and EVs treatment entirely reversed the reduction (Figure 2(a)). To further explore the mechanism of miR-30 reversion, we used specific miR-30 antagomir to treat MSCs. MSCs were cocultured with the specific antagomir at a concentration of 50 nM for 24 h. After that, we changed the culture solution for another 24 h before isolation of EVs. Data showed that miR-30b/c/d were, respectively, absent in EVs derived from MSCs (Figure 2(b)). Meanwhile, antagomir-treated EVs group revealed lower miR-30 expression as well as the vehicle group (Figure 2(c)). The absence of miR-30 in EVs canceled the miR-30 restoration effects in normal EVs treatment group. In addition, the mature sequence of human miR-30b/c/d was the same as the rats'. The same phenomenon could also be observed in renal tubular epithelial cells in vitro (Supplement 1 in Supplementary Material available online at http://dx.doi.org/10.1155/2016/2093940). Furthermore, at 3 hours after the injection to the injured rats, some EVs were detected within tubules (Supplement 2). In conclusion, hWJMSC-EVs may deliver exogenous miRNA to injured renal tubular epithelial cells, which may be an important pathway that EVs affect.

### 3.3. EVs-Derived miR-30 Members Regulate Mitochondrial Fission through DRP1

**Figure 3:**
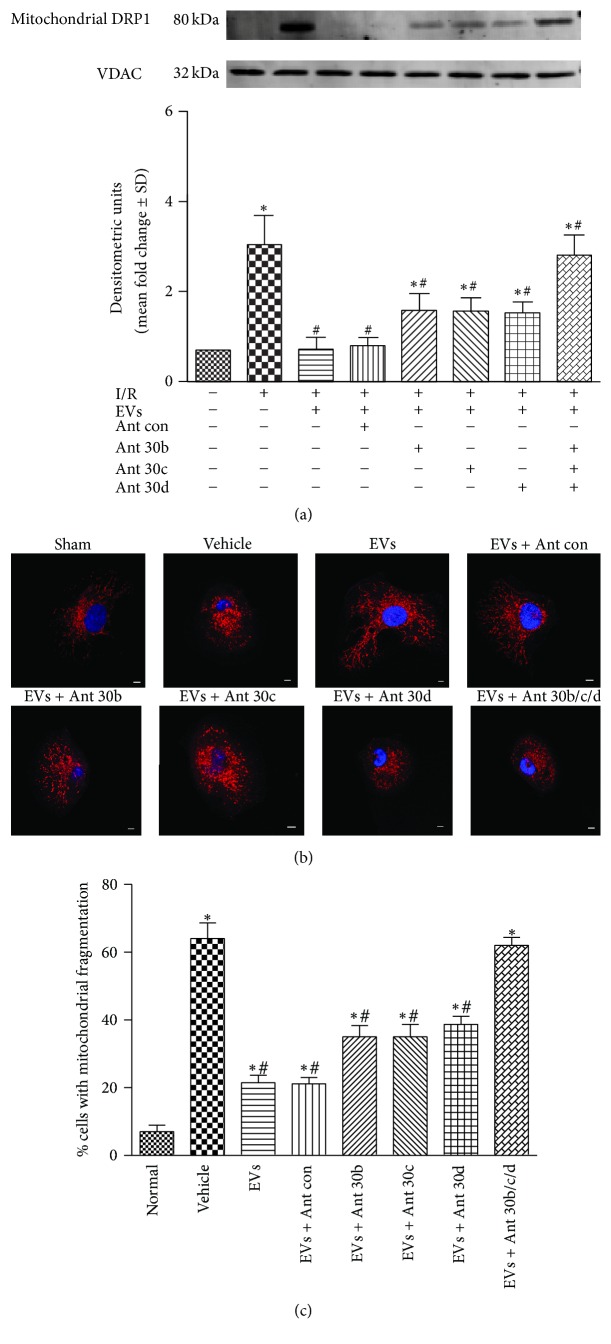
EVs-derived miR-30 members regulate mitochondrial fission through DRP1. (a) Mitochondrial DRP1 expression in kidney tissues in different experimental conditions, including sham, vehicle, EVs, and miR-30b/c/d antagomir-treated EVs group. VDAC antibody served as a loading control. (b) The morphological alterations of mitochondria in renal tubular epithelial cells treated in these groups. (c) The percentage of cells undergoing mitochondrial fission. Data in (a) and (c) are presented as mean ± SD; *n* = 6; ^*∗*^ *P* < 0.05, versus sham; ^#^ *P* < 0.05, versus vehicle.

To understand how hWJMSC-EVs exert effects on mitochondrial fission, we test whether EVs-derived miR-30 family plays a crucial role in regulating mitochondrial fission through DRP1. In rat kidneys, enforced DRP1 expression and activation were seen in antagomir-treated EVs group, especially in miR-30b/c/d cotreated EVs group (Figure 3(a)). Mitochondrial DRP1 showed high expression, respectively, in vehicle group and antagomir-treated EVs group.

As we expected, mitochondrial fragmentation was also increased in antagomir-treated EVs group in vitro (Figure 3(b)). Mitochondria of renal tubular cells were filamentous or of thread-like appearance in normal and EVs treatment group. However, the antagomir-treated group showed more round fragments of mitochondria as the vehicle group. Quantification of mitochondrial fragmentation is shown in Figure 3(c). These data suggested a role of miR-30b/c/d in EVs treatment of IRI.

### 3.4. Mitochondrial Apoptotic Pathways Are Inhibited by EVs-Derived miR-30

**Figure 4:**
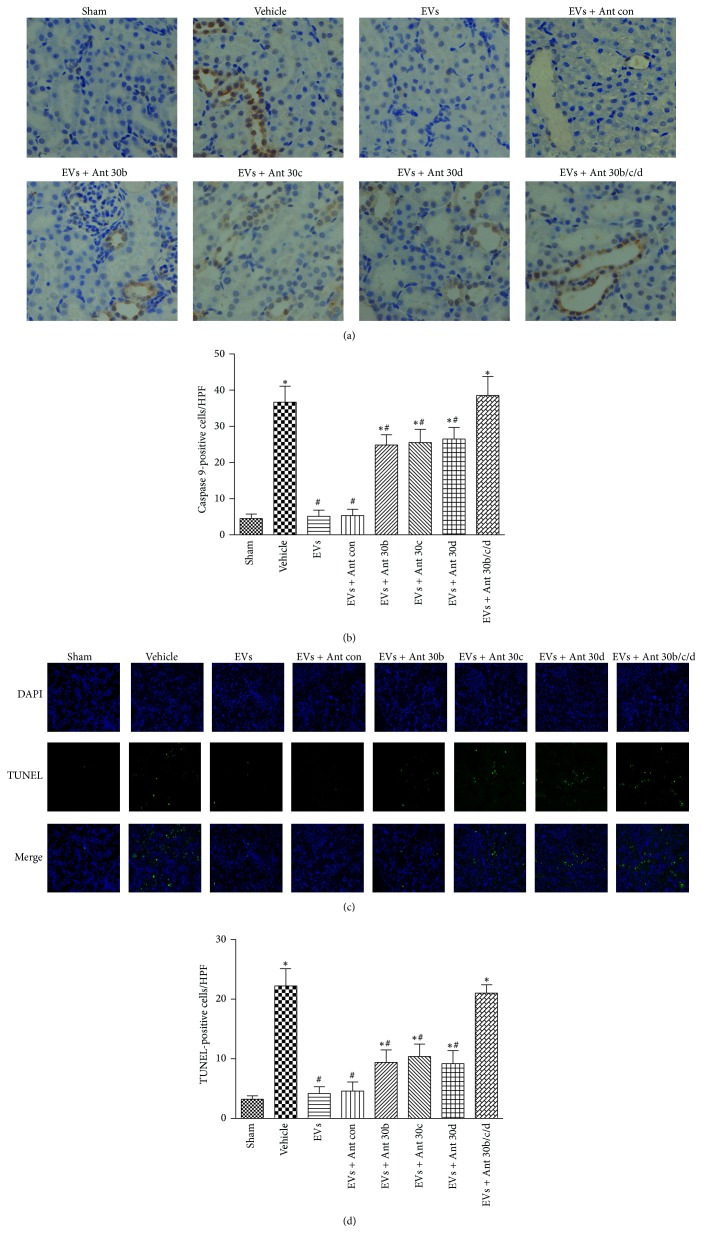
Mitochondrial apoptotic pathways are inhibited by EVs-derived miR-30. Immunochemistry staining for caspase 9 and TUNEL assay were carried out in kidney tissue. (a) Representative micrographs illustrating caspase 9 expression in kidney tissues. In comparison with EVs group, the vehicle group and antagomir-treated group exhibited stronger positive staining for caspase 9 in kidney tissue sections (the original magnification is 400x). (b) Quantification of caspase 9-positive tubular epithelial cells in the kidney sections. (c) Representative images of TUNEL staining in kidney tissues (magnification 400x). (d) Quantification of TUNEL-positive tubular epithelial cells in the kidney sections. Data in (b) and (d) are presented as mean ± SD; *n* = 6; ^*∗*^ *P* < 0.05, versus sham; ^#^ *P* < 0.05, versus vehicle.

Mitochondrial fragmentation has been observed in models of AKI, and this process contributes to the resulting apoptosis. Then, we evaluated mitochondrial apoptotic pathway in our AKI models. As shown in Figures 4(a) and 4(b), immunohistochemistry revealed that caspase 9 expression was enhanced in vehicle group yet reduced in EVs treatment group. However, miRNA antagomir abolished the effect.

TUNEL assay was next executed to further confirm the renal tubular cells apoptosis (Figures 4(c) and 4(d)). The number of TUNEL-positive cells significantly increased at 24 h after IRI compared to the sham group, while EVs treated group exhibited fewer TUNEL-positive cells than the vehicle group and miRNA antagomir alleviated this effect. Taken together, it appears that EVs-derived miR-30 can block the mitochondrial apoptotic pathways.

### 3.5. Protective Effect of EVs in Renal Function

**Figure 5:**
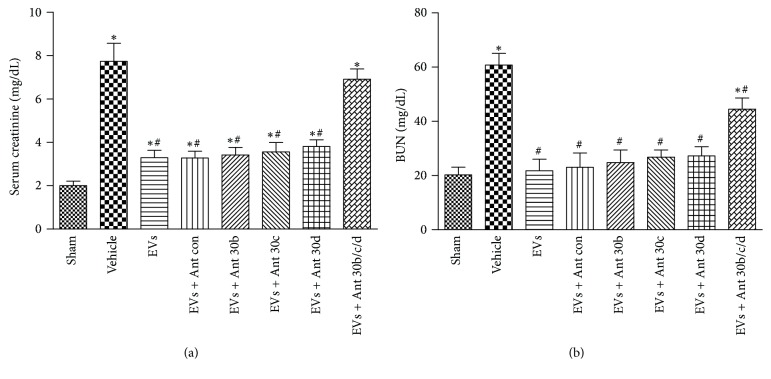
Protective effect of EVs in renal function. (a, b) Serum creatinine and BUN levels were measured at 24 h after injury in the sham, vehicle, EVs, and antagomir-treated EVs groups. Data in (a) and (b) are presented as mean ± SD; *n* = 6; ^*∗*^ *P* < 0.05, versus sham; ^#^ *P* < 0.05, versus vehicle.

We evaluated the effects of hWJMSC-EVs in the recovery of renal function in IRI model (Figures 5(a) and 5(b)). In comparison with sham-operated animals, rats subjected to kidney IRI showed a significant rise in serum creatinine and blood urea nitrogen (BUN). EVs treatment abrogated the injury and miRNA antagomir did not block this effect in miR-30b/c/d single inhibited group. This may be because the residual nephron could maintain the renal function temporarily.

## 4. Discussion

Here we have identified a miR-30-related antiapoptotic pathway involving DRP1 and mitochondria, which may be one of the mechanisms by which hWJMSC-EVs alleviate renal ischemia reperfusion injury. Our data showed that hWJMSC-EVs could enhance miR-30b/c/d of renal tubular cells and mitigate the activation of DRP1 and mitochondrial fragmentation which leads to antiapoptotic effects.

It is generally thought that the therapeutic effects of MSCs administration were due to endocrine or paracrine mechanisms [17, 23]. Extracellular vesicles derived from MSCs are a key instrument of cell-cell communication [19, 24–27]. EVs carry proteins, cytokines, chemokines, growth factors, and RNA content to the target cells so as to modulate proliferation, angiogenesis, and apoptosis [26, 28]. Our group also found that hWJMSCs-EVs could alleviate acute and chronic kidney injury through anti-inflammatory and antioxidative pathways [15, 21, 22]. MicroRNAs, one of the crucial genetic pieces of information coming from EVs, got more and more emphasis these days [29, 30]. Lindoso et al. demonstrated that EVs not only directly transfer miRNA in injured PTECs but also have a transcriptional modulation function [31]. The present study demonstrated that hWJMSCs-EVs could carry miRNAs to rat kidney, participating in kidney damage repair.

The miR-30 family is involved in several cellular processes, including cardiomyocytes exposed to oxidative stress or ischemia injury and apoptosis of type II alveolar epithelial cells [20, 32, 33]. In the present study, we used a rat model of unilateral IRI to determine whether miRNAs play a central role in the treatment of EVs. Our data demonstrate that miR-30b/c/d reduced in renal tubular cells during IRI and hWJMSCs-EVs could increase the expression of miR-30b/c/d in injured tubular cells both in vitro and in vivo. The transfer effect of EVs to injured renal tubular cells has been supported not only by in vitro cell culture and in vivo animal studies [25, 34] but also by our previous finding [21]. Then, we treated hWJMSCs with miR-30 antagomir. It successfully eliminated the corresponding miRNA in hWJMSCs-EVs and renal recovery effect, further confirming the miRNA transfer and its therapeutic effect.

Mitochondrial fission is observed in IRI and is strongly linked with the onset and development of renal injury. To explore the potential mechanism of EVs in the IRI, we detected the mitochondrial fission related protein DRP1. Our results show that the DRP1 was activated rapidly following acute kidney cell injury and translocated to mitochondria leading to mitochondrial fragmentation, which is in accordance with the published data [35]. The EVs treatment suppressed the activation of DRP1 and mitochondrial fission. Mitochondrial fission is one of the initial factors that trigger the apoptosis pathway. Further, the EVs treatment showed antiapoptosis effect and protected the injured kidney. As we expected, miR-30 antagomir mitigated this effect, especially in miR-30b/c/d antagomir cotreated group. We observed almost the same degree of injury as the vehicle group, including the activation of DRP1, mitochondrial fragmentation, TUNEL staining, caspase 9 staining, and renal function. Thus, a miRNA may have multiple targets and different miRNAs may have the same target. What is more, sometimes treatment focus on a target miRNA may have limited function. We supposed that the function of EVs may be dependent on this synergistic effect of miRNAs.

## 5. Conclusion

Our research reveals links among EVs, miR-30, and DRP1 in the apoptotic program of the kidney. Future studies are needed to elucidate how this pathway is integrated into complex apoptotic cascades and elucidate its relationship with mitochondrial fission. Our results suggest that modulation of miR-30 from EVs may represent a therapeutic approach to treat apoptosis-related renal ischemia reperfusion injury.

## Supplementary Material

S1 MiR-30b/c/d levels in renal tubular epithelial cells in different experimental conditions, including normal, vehicle, EVs and miR-30b/c/d antagomir treated EVs group. The absence of miR-30 in EVs canceled the miR-30 restoration effects in normal EVs treatment group in vitro. S2 Here we performed an EVs tracker experiment to show more evidence that EVs can transfer their contents to the renal tubular epithelial cells. The IRI model was as described in the article. Before EVs injection, we used PKH26 Red Fluorescent Cell Linker (Sigma) to label EVs. In briefly, after the first ultracentrifugation, we used a 1uM labeling solution to resuspend the precipitation and incubate for 20 minutes at room temperature. Then the EVs were submitted to a second ultracentrifugation in the same conditions. Next steps were as same as we mentioned before. And we collected the kidney for frozen section at 3, 12 and 24 hours. EVs were detectable in the tubular epithelial cells at 3 hours，which could further proved the transfer of EVs in kidney repair.

## Abbreviations

IRI: Ischemia reperfusion injury
EVs: Extracellular vesicles
hWJMSCs: Human Wharton Jelly mesenchymal stromal cells
AKI: Acute kidney injury
DRP1: Dynamin-related protein 1.
