# Supplementary figures and images for: Mesenchymal Stromal Cells Derived Extracellular Vesicles Ameliorate Acute Renal Ischemia Reperfusion Injury by Inhibition of Mitochondrial Fission through miR-30
Source: Stem Cells Int. 2016 Oct 5;2016:2093940. doi: 10.1155/2016/2093940 (PMC5069372; doi:10.1155/2016/2093940)

611 Figure.S1

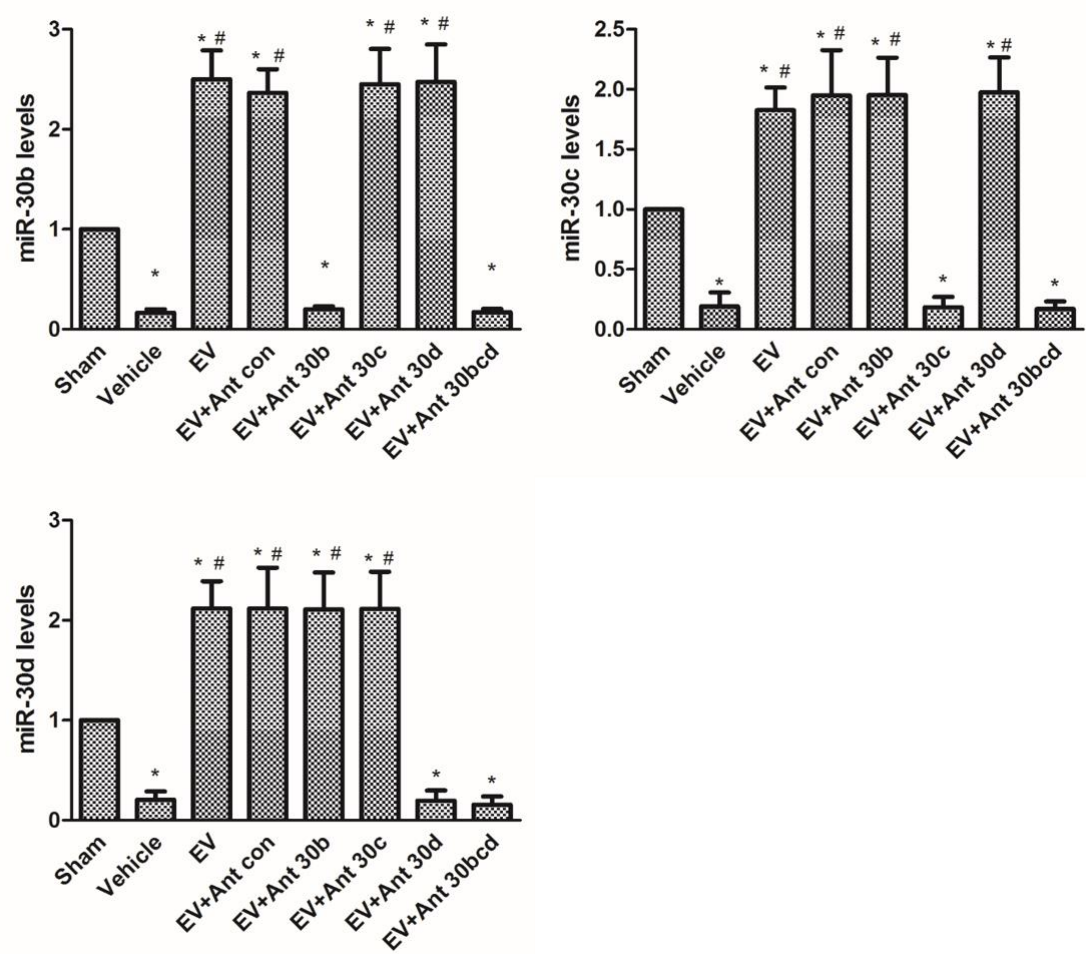

612

613 Figure.S2

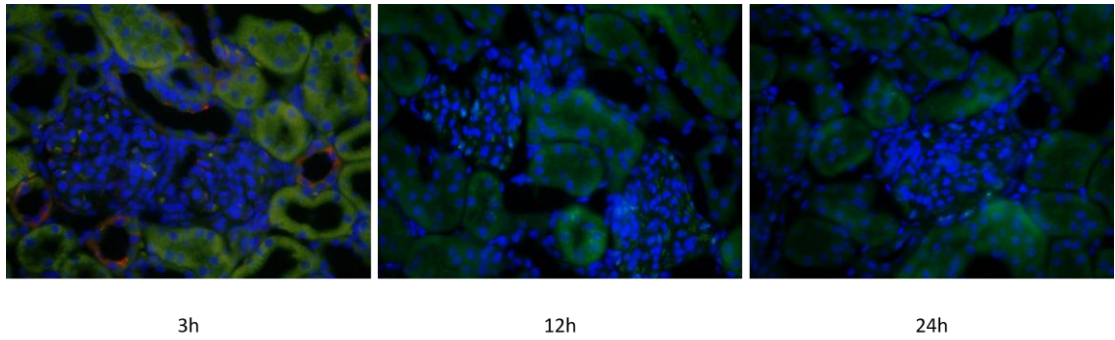

614

Supplement: Supplementary file 1 — S1 MiR-30b/c/d levels in renal tubular epithelial cells in different experimental conditions, including normal, vehicle, EVs and miR-30b/c/d antagomir treated EVs group. The absence of miR-30 in EVs canceled the miR-30 restoration effects in normal EVs treatment group in vitro. S2 Here we performed an EVs tracker experiment to show more evidence that EVs can transfer their contents to the renal tubular epithelial cells. The IRI model was as described in the article. Before EVs injection, we used PKH26 Red Fluorescent Cell Linker (Sigma) to label EVs. In briefly, after the first ultracentrifugation, we used a 1uM labeling solution to resuspend the precipitation and incubate for 20 minutes at room temperature. Then the EVs were submitted to a second ultracentrifugation in the same conditions. Next steps were as same as we mentioned before. And we collected the kidney for frozen section at 3, 12 and 24 hours. EVs were detectable in the tubular epithelial cells at 3 hours，which could further proved the transfer of EVs in kidney repair. [file 2093940.f1.pdf]
